# Text-Book of Diseases of the Kidneys and Genito-Urinary Organs

**Published:** 1899-03

**Authors:** 


**Text-Book of Diseases of the Kidneys and Genito-Urinary Organs.** By Prof. Dr. PAUL FÜRBRINGER. Translated by W. H. GILBERT, M.D. Vol. II. Pp. vi., 310. London: H. K. Lewis. 1898.

The first volume of this work, which dealt with the medical aspect of the subject, was favourably reviewed by us in March, 1896. Of the second volume, which considers the surgical diseases of the organs in question, it is not possible to speak highly. In fact, the diseases of the genito-urinary organs are now so specialised that it is impossible for one man to deal satisfactorily with the whole of them. It would have been better if the author had confined his information to the diagnosis and medical treatment of those diseases of the urinary organs which fall to the lot of the physician.

The bibliography seems an important feature, but it is arranged alphabetically only according to the authors' names, and not according to subjects. It seems to us that, with Mr. Jacobson's work on diseases of the male genital organs and Mr. Henry Morris's recent book on diseases of the urinary organs, and the very full consideration of the subject in our larger text-books of surgery, there was really no need for the translation of Professor Fürbringer's second volume. To English readers some of the terms used are rather remarkable. For instance, hydrocele in connection with epididymitis, which we should call an acute hydrocele, is spoken of as periorchitis acuta.

The author regrets that pain in the groin is not recognised as common in acute epididymitis. We should certainly have considered that it was known to every observant student. The diagnosis of renal calculus from the other morbid conditions which may readily be mistaken for it receives most inadequate notice, and the reference to the surgical treatment of renal disease is very imperfect and unsatisfactory. Much space is given to an enumeration of the various futile attempts which have been made to discover the condition of the separate kidneys, and yet there is no mention of the work of Howard Kelly in this direction.

We cannot recommend the work either as a concise statement of the leading facts of genito-urinary surgery, or as any approach to an exhaustive treatise on the subject. Some of the information given we even regard as dangerous. For instance, in speaking of the diagnosis of renal cancer, we are told of a case in which aspiration was not only used, but repeated several times for diagnosis, and then after death it was found that the growth was in the "small omentum" and not in the kidney at all! Surely such proceedings are highly dangerous and not to be recommended by illustrative cases. Then again, in speaking of the treatment of stone in the bladder, we are told that the bladder has been opened from the rectum, as if such a proceeding was an approved method of operation.

